# Eight-week dietary methionine restriction does not impair resistance exercise-induced mTORC1 signalling activation in rats

**DOI:** 10.1016/j.bbrep.2026.102473

**Published:** 2026-01-31

**Authors:** Naoki Fukao, Mito Watanabe, Ryo Takagi, Koki Okumura, Naomi Yoshii, Koma Kawabata, Satoshi Fujita

**Affiliations:** a Graduate School of Sport and Health Science, Ritsumeikan University, Kusatsu, Shiga, 525-8577, Japan; b Ritsumeikan Global Innovation Research Organization, Ritsumeikan University, Kusatsu, Shiga, Japan; c School of Nursing and Rehabilitation Sciences, Showa University, Yokohama, Japan; d Research Organization of Science and Technology, Ritsumeikan University, Kusatsu, Shiga, Japan; e Faculty of Sport and Health Science, Ritsumeikan University, Kusatsu, Shiga, Japan

**Keywords:** Essential amino acids, Muscle protein synthesis, mTORC1, Muscle protein degradation, Muscle contraction, Malnutrition

## Abstract

Essential amino acids (EAA) and resistance exercise (RE) are well-known approaches to activate muscle protein synthesis. Methionine is an EAA that stimulates mechanistic/mammalian target of rapamycin complex 1 (mTORC1) signalling. It is believed that the lack of a single EAA could blunt protein synthesis. However, to our knowledge, no study has investigated the effects of methionine-restricted diet (MR) on RE-induced anabolic and catabolic signalling in skeletal muscle. Therefore, in this study, we aimed to investigate the effects of MR on RE-induced muscle protein synthesis and breakdown-related signalling. Male Sprague–Dawley rats were randomly divided into Control and MR group, and rats in the MR group were fed the experimental diets for 8 weeks. After the dietary intervention, RE was performed. p70S6K phosphorylation exhibited a significant main effect of RE and MR, with higher values observed across both conditions. FoxO3a Ser253 phosphorylation showed a significant main effects of both RE and MR, with lower values observed across conditions, while MuRF-1 protein expression showed a significant main effect of MR, with lower values observed in the MR condition. Furthermore, muscle protein synthesis demonstrated a significant main effect of MR, with higher values observed across MR conditions. These results suggested that the MR for 8 weeks neither attenuate RE-induced muscle anabolic response nor enhance catabolic response in rat skeletal muscle.

## Highlights

• Methionine-restricted diet was found to activate mTORC1 signalling and muscle protein synthesis.
• Methionine-restricted diet did not attenuate anabolic response to resistance exercise.
• Methionine-restricted diet decreased catabolic markers, even when combined with resistance exercise.

## Graphical abstract

**Figure:**
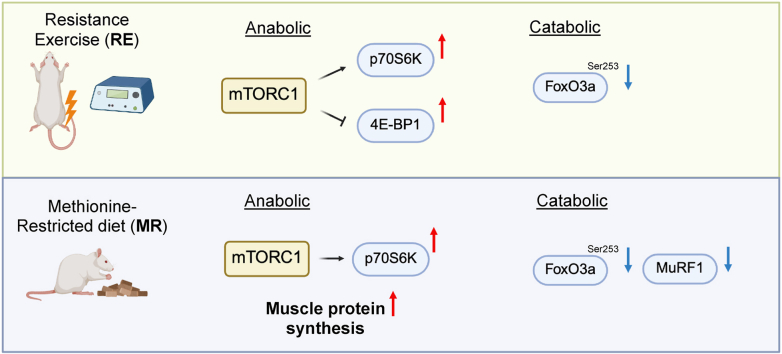

## Abbreviations

EAA: Essential amino acids
mTORC1: Mechanistic/mammalian target of rapamycin complex 1
SAM: S-adenosylmethionine
MR: Methionine-restricted diet
RE: Resistance exercise
TBS-T: Tris-buffered saline containing 0.1 % Tween 20

## 1 Introduction

Skeletal muscle mass is regulated by protein synthesis and breakdown [1]. Exercise and exogenous essential amino acids (EAA) dramatically change muscle protein metabolism [[2], [3], [4]], and both long-term interventions are beneficial for muscle mass [5,6].

Muscle protein synthesis is primarily regulated by the mechanistic/mammalian target of rapamycin complex 1 (mTORC1), and amino acid-induced muscle protein synthesis is increased via mTORC1 signalling [7]. Methionine and S-adenosylmethionine (SAM), which is generated by methionine and adenosine, work to provide a methyl group to DNA and RNA [8]. Methionine stimulated the mTORC1 downstream proteins in C2C12 [9]. Moreover, SAM is a key regulator that disrupts the SAMTOR-GATOR1 complex, which is a negative regulator of mTORC1 [10]. Therefore, methionine and its product SAM activate mTORC1 signalling. Methionine is primarily present in animal-derived proteins; therefore, individuals with a strict plant-based diet are at risk of methionine deficiency [11]. Even though recent studies have focused on the effects of plant-based protein and diets on muscle protein synthesis [12,13], the specific effects of restricting each EAA remain largely unknown. Some studies have investigated the effects of daily methionine-restricted diet (MR) on skeletal muscle. Mice fed MR for 5 weeks showed increased muscle mass compared to that of control mice [14]. However, muscle protein synthesis of mixed and cytosolic fraction was significantly lower than that in the control mice [14]. Another study using fish muscle reported that the MR inhibited mTORC1 signalling and activated the autophagy-lysosomal and ubiquitin-proteasome systems [15]. However, different patterns of skeletal muscle growth observed in fish and rodents throughout their lives may lead to different outcomes [16,17]. Furthermore, MR often demonstrates its effects when combined with a high-fat diet, and the impact of pure MR on skeletal muscle protein metabolism and molecular signalling remain poorly understood [18,19].

Resistance exercise (RE) is the most effective exercise for enhancing muscle protein synthesis and chronic training induces muscle hypertrophy [2,3,20]. RE enhances the mTORC1 pathway, which initiates translation [2]. To facilitate muscle protein synthesis, EAA is crucial as it is a substrate for muscle protein. Protein synthesis is thought to be highly associated with EAA because an imbalance in the EAA profile affects it [21]. However, no studies have investigated whether a single restricted EAA attenuates RE-induced muscle protein synthesis. A previous study using synergistic ablation, which induced super-physiological muscle hypertrophy in rodents, showed that MR enhanced muscle mass in old mice with synergistic ablation [19]. However, the underlying mechanism has not yet been elucidated [19]. In particular, mTORC1-downstream signalling and protein degradation pathway remain unclear.

Therefore, we aimed to investigate the impacts of MR on RE-induced responses in rat skeletal muscle. We hypothesised that dietary MR impairs RE-induced anabolic response in rat skeletal muscle.

## 2 Methods

### 2.1 Animals and experimental design

This study was approved by the Ritsumeikan University Animal Experimentation Committee (BKC2024-009) and conducted in accordance with the Declaration of Helsinki. Twelve 10-week-old male Sprague–Dawley rats were purchased from Shimizu Laboratory Materials. We did not use female-rats to avoid sex-differences for homocysteine level which could affect methionine metabolism as previous studies shown [22]. All rats were maintained at 23 ± 1 °C with a 12-h light/dark cycle and provided water and food *ad libitum*. After one week of environmental acclimatization, the rats were randomly divided into Control or MR group. Each diet was provided for eight weeks. After eight weeks, the rats were fasted overnight. The rats were anaesthetised with isoflurane, and RE was conducted. 6 hours after RE, the rats were euthanized, and the tissues were harvested and stored in LN_2_. Tissues were stored at– 80 °C until analysis.

### 2.2 Dietary intervention

Rats in Control group were fed an AIN-93G diet (Methionine: 0.510 g/100 g diet, Oriental Yeast Co., Ltd., Tokyo, Japan), while rats in MR group were fed an AIN-93G diet (Methionine: 0.102 g/100 g diet, Oriental Yeast Co., Ltd.) for 8 weeks. Previous studies reported that MR for 8 weeks affected skeletal metabolic profile, especially mTORC1-related proteins in mice [23]. Thus, we assumed that 8 weeks is adequate duration to observe muscle adaptation by MR and to confirm the combined effects with RE. Details of each diet are shown in Table 1.

**Table 1:** Diet composition. Con: Control. MR: Methionine-restricted diet.

	Con	MR
Net energy, kcal/100g	368.2	368.2
Protein, %	18.2	17.9
Fat, %	7.1	7.1
Carbohydrate, %	57.8	58.2
Methionine, %/100g	0.51	0.102
Amino acids/100g	18.6	18.2

### 2.3 RE protocol

RE was performed as previously described [2,24,25]. Briefly, under isoflurane inhalation anaesthesia, the right leg was shaved and exposed skin was cleaned with ethanol. The rats were then placed in the supine position, and the right leg was placed on a footplate and fixed such that the ankle joint angle was 90°. The gastrocnemius muscle of the right leg was stimulated percutaneously using electrodes (Vitrode V, Ag/AgCl; Nihon Kohden, Tokyo, Japan) connected to an electrical stimulator (SEN-0823; Nihon Kohden) and an isolator (SS-104j; Nihon Kohden). The frequency of electrical stimulation was set at 100 Hz, and the voltage was adjusted to exert maximum isometric contraction. Five sets of electrical stimulation were performed, each consisting of 10 cycles of 3 s of electrical stimulation with a 7-s interval between each set. There was a 3-min rest between sets. The right gastrocnemius of each rat was treated as the exercise leg, and the left gastrocnemius was treated as the sedentary leg. This rat RE model is capable of eliciting acute responses and chronic skeletal muscle adaptations in rats that are comparable to those reported in human resistance exercise studies [2,20,26].

### 2.4 Western blotting

Western blotting was performed as previously described, with minor modifications [25]. Briefly, frozen muscle samples were powdered and homogenised in RIPA buffer (Cell Signaling Technology (CST), Danvers, MA, USA) containing a protease inhibitor cocktail (Sigma-Aldrich, St. Louis, MO, USA) and a phosphatase inhibitor cocktail (Sigma-Aldrich). After centrifugation of the homogenate (10000×*g*, 10 min, 4 °C), the protein concentration of the collected supernatant was determined using a Protein Assay BCA kit (Nacalai Tesque, Kyoto, Japan). Then, 3 × Blue Loading Buffer, DTT (CST), supernatant, and distilled water were mixed, and the mixture was boiled at 95 °C for 5 min. Each sample was separated by electrophoresis on acrylamide gel with the same amount of protein and transferred onto PVDF membranes (Bio-Rad, Hercules, CA, USA). The membranes were washed with Tris-buffered saline containing 0.1 % Tween 20 (TBS-T) for 5 min and blocked with TBS-T containing 5 % skim milk for 60 min at room temperature. After blocking, the membrane was washed in TBS-T and incubated overnight (4 °C) with primary antibody The following antibodies were used: p70S6K(#34475, CST), Phospho-p70S6K Thr389 (#9205, CST), rpS6 (#2217, CST), Phospho-rpS6 Ser240/244 (#2215, CST), 4E-BP1 (#9644, CST), Phospho-4E-BP1 Thr37/46 (#2885, CST), Puromycin (#MABE343, Merck Millipore, Burlington, MA, USA), Akt (#4691, CST), Phospho-Akt Thr308 (#13038, CST), Phospho-Akt Ser473 (#9271, CST), AMPKα (#2532, CST), Phospho-AMPKα Thr172 (#2537, CST), ULK1 (#8054, CST), Phospho-ULK1 Ser317 (#12753, CST), LC3B (#2775, CST), p62 (#PM045, Medical & Biological Laboratory, Nagoya, Japan), FoxO3a (#2497, CST), Phospho-FoxO3a Ser253 (#13129, CST), Fbx32 (#168372 Abcam, Cambridge, UK), MuRF-1 (#sc-398608, Santa Cruz Biotechnology, Dallas, TX, USA). The following day, the membrane was washed with TBS-T and the appropriate secondary antibody was added to TBS-T containing 1–3 % skim milk and incubated at room temperature for 1 h. The membrane was washed with TBS-T for 5 min and the bands were detected using Luminata Forte Western HRP Substrate (Millipore, CA, USA) with FUSION Chemiluminescence Imaging System (M&S Instruments, Osaka, Japan). Band intensities were calculated by standardizing the quantified value of the amount of protein applied to each lane by Ponceau S staining using ImageJ software (ver 1.53k; National Institutes of Health, Bethesda, MD, USA).

### 2.5 Muscle protein synthesis

Muscle protein synthesis was measured using the SUnSET method [27]. Puromycin diluted in PBS (0.04 μmol/g body weight) was injected intraperitoneally in each rat 15 min before sacrifice under isoflurane anaesthesia. Powdered muscle samples were mixed with RIPA buffer, and the supernatant was obtained after centrifugation (2000×*g*, 3 min, 4 °C). The procedure was the same as that used for Western blot analysis.

### 2.6 Plasma amino acids analysis

The procedure was performed as previously described [28]. Blood samples with 10 % EDTA were centrifuged at 1700×*g* for 10 min at 4 °C then plasma was collected. Plasma samples were mixed with 15 % sulfosalicylic acid and incubated on ice for 20 min. The samples were centrifuged at 7000×*g* for 10 min at 4 °C. The supernatant was injected into ultrafiltration filter (UFC501096, Millipore) and re-centrifuged at 14000×*g* for 60 min at 4 °C. The lower layers were collected and taken as samples after protein removal [29,30]. Amino acid concentrations were analysed using a high-speed analyzer (L-8900; Hitachi, Tokyo, Japan). Amino acids were separated by ion exchange chromatography and detected spectrophotometrically after post-column reaction with ninhydrin.

### 2.7 Statistical analysis

All values are expressed as mean ± SE. IBM SPSS Statistics (ver. 29; SPSS Inc., Chicago, IL, USA) was used for statistical analysis. Data were analysed using a two-way analysis of variance (RE × MR), and multiple comparisons by Bonferroni were performed only when a significant interaction was found. The other parameters were analysed using unpaired t-tests. The significance level was set at *p* < 0.05.

## 3 Results

### 3.1 Characteristics of the animals

Body weights were not significantly different between the two groups (Table 2). No significant differences in food intake were observed during the intervention period (Table 2). The muscle wet weight mass of gastrocnemius in sedentary leg was not different between the groups (Table 2). Plasma methionine concentration did not differ significantly between the two groups. Similar results were observed for the other amino acids (Table 2).

**Table 2:** Characteristics of the animals (n = 6/group). Con: Control. MR: Methionine-restricted diet. Data are expressed as mean ± SE.

	Con	MR	*P*-value
Body weight, g	445.5 ± 17.9	435.9 ± 11.0	0.658
Food intake, g/day	18.2 ± 1.0	20.1 ± 0.7	0.312
Gastrocnemius muscle, mg/BW	5.07 ± 0.11	5.06 ± 0.15	0.977
Isoleucine, μM	189.5 ± 14.9	197.6 ± 20.6	0.756
Leucine, μM	298.0 ± 21.5	315.3 ± 30.4	0.652
Valine, μM	353.3 ± 25.9	373.9 ± 32.7	0.632
Phenylalanine, μM	115.7 ± 18.4	121.9 ± 19.6	0.584
Tryptophan, μM	134.8 ± 13.0	147.1 ± 12.0	0.503
Methionine, μM	90.3 ± 8.0	93.7 ± 6.5	0.755
Histidine, μM	125.2 ± 6.9	124.3 ± 7.6	0.937
Lysine, μM	526.8 ± 49.6	535.1 ± 42.8	0.902
Threonine, μM	509.8 ± 36.6	561 ± 45.5	0.401
3-methylhistidine, μM	8.45 ± 0.4	8.91 ± 0.7	0.544

### 3.2 mTORC1-related proteins and muscle protein synthesis

**Fig. 1:**
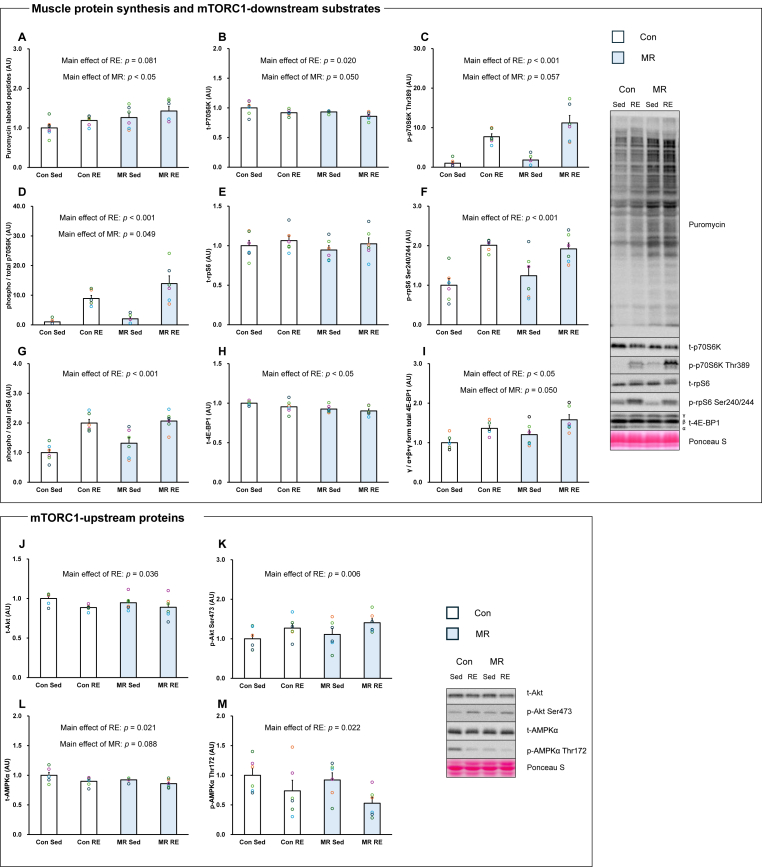
Effects of resistance exercise and methionine-restricted diet on muscle protein synthesis and mTORC1 signalling-related proteins (n = 6/group). A: Puromycin labelled peptides. B: total-p70S6K. C: phosphorylation-p70S6K Thr389. D: phosphorylation ratio of p70S6K. E: total-rpS6. F: phosphorylation-rpS6 Ser240/244. G: Phosphorylation ratio of rpS6. H: total-4E-BP1. I: total-4E-BP1 γ form ratio. J: total-Akt. K: phosphorylation-Akt Ser473. L: total-AMPK. M: phosphorylation-AMPK Thr172. Con: Control. Sed: Sedentary. RE: Resistance exercise. MR: Methionine-restricted diet. Data are expressed as mean ± SE.

Total p70S6K protein abundance showed a significant main effect of RE, with lower values observed in the RE condition, and a borderline main effect of MR, with no interaction between factors (Main effect of RE: *p* < 0.05, Main effect of MR: *p* = 0.050, Fig. 1B). p70S6K Thr389 phosphorylation exhibited a significant main effect of RE, with higher values observed in the RE condition, and a trend toward a main effect of MR, with no interaction (Main effect of RE: *p* < 0.001, Main effect of MR: *p* = 0.057, Fig. 1C). Similarly, the phosphorylation rate of p70S6K exhibited significant main effects of both RE and MR, with higher values observed across conditions for each factor and no interaction (Main effect of RE: *p* < 0.001, Main effect of MR: *p* = 0.049, Fig. 1D). Total rpS6 protein expression was not significantly affected by either RE or MR. In contrast, rpS6 Ser240/244 phosphorylation and the phosphorylation rate of rpS6 showed significant main effects of RE, with higher values observed in the RE condition and no interaction (Main effect of RE: *p* < 0.001 respectively, Fig. 1F and G). Total 4E-BP1 protein expression showed a significant main effect of MR, with lower values observed in the MR condition, whereas no main effect of RE was detected (Main effect of RE: *p* = 0.152, Main effect of MR: *p* < 0.05, Fig. 1H). The γ form of total 4E-BP1 exhibited a significant main effect of RE, with higher values observed in the RE condition, and a borderline main effect of MR, with no interaction (Main effect of RE: *p* < 0.05, Main effect of MR: *p* = 0.050, Fig. 1I).

Total Akt protein showed a significant main effect of RE, with lower values observed in the RE condition, and no main effect of MR (Main effect of RE: *p* < 0.05, Main effect of MR: *p* = 0.528, Fig. 1J). Akt Ser473 exhibited a significant main effect of RE, with higher values observed in the RE condition, whereas no main effect of MR was detected (Main effect of RE: *p* < 0.05, Main effect of MR: *p* = 0.320, Fig. 1K). Total AMPK protein expression exhibited a significant main effect of RE, with lower values observed in the RE condition, and a trend toward a main effect of MR, with no interaction (Main effect of RE: *p* < 0.05, Main effect of MR: *p* = 0.088, Fig. 1L). AMPK Thr172 phosphorylation showed a significant main effect of RE, with lower values observed in the RE condition, and no main effect of MR (Main effect of RE: *p* < 0.05, Main effect of MR: *p* = 0.288, Fig. 1M). Muscle protein synthesis exhibited a trend toward a main effect of RE and a significant main effect of MR, with higher values observed across conditions in the presence of MR, and no interaction between factors (Main effect of RE: *p* = 0.081, Main effect of MR: *p* < 0.05, Fig. 1A).

### 3.3 Muscle protein-degradation pathway

**Fig. 2:**
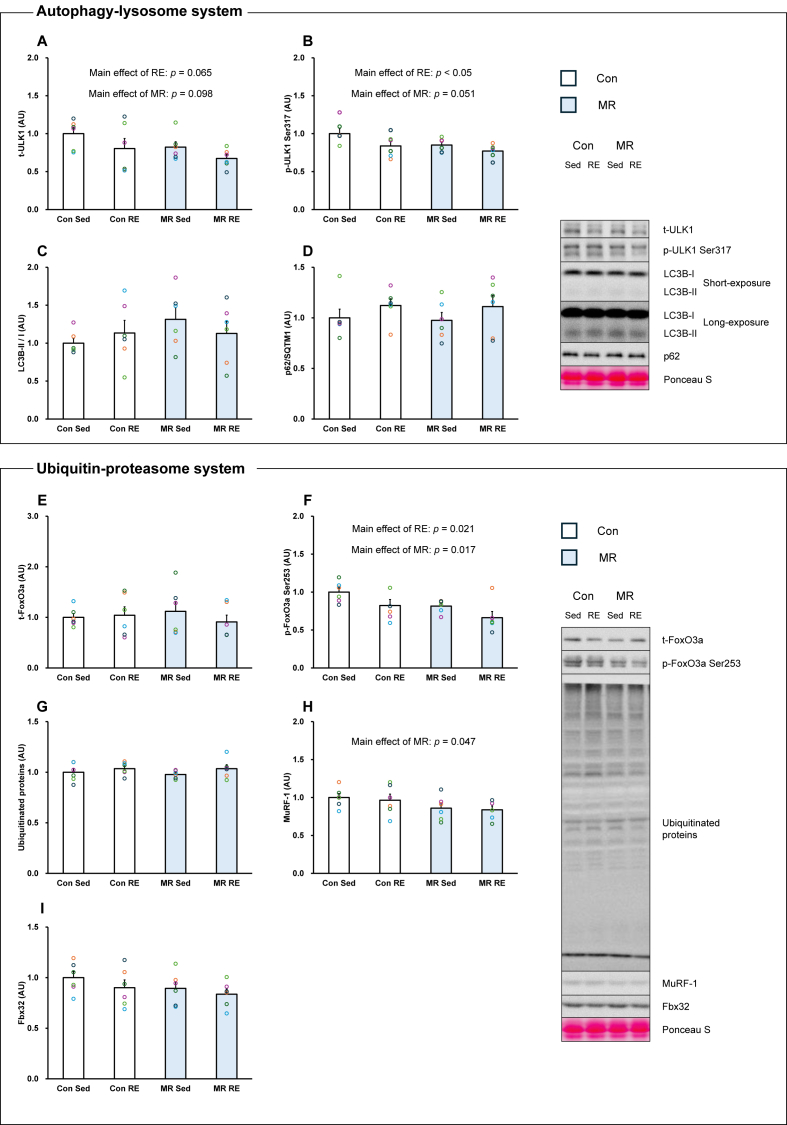
Effects of resistance exercise and methionine-restricted diet on the catabolic-related factors (n = 6/group). A: total-ULK1. B: phosphorylation-ULK1 Ser317. C: LC3B-II/I ratio. D: p62. E: total-FoxO3a. F: phosphorylation-FoxO3a Ser253. G: Ubiquitinated proteins. H: MuRF-1. I: Fbx32. Con: Control. Sed: Sedentary. RE: Resistance exercise. MR: Methionine-restricted diet. Data are expressed as mean ± SE.

Total ULK1 protein expression showed trends toward main effects of both RE and MR, with lower values observed across conditions, and no interaction between factors (Main effect of RE: *p* = 0.065, Main effect of MR: *p* = 0.098, Fig. 2A). ULK1 Ser317 phosphorylation showed a significant main effect of RE, with lower values observed in the RE condition, and a trend toward a main effect of MR, with no interaction (Main effect of RE: *p* < 0.05, Main effect of MR: *p* = 0.051, Fig. 2B). LC3B-II/LC3B–I ratio and p62 protein expression were not significantly affected by either factor (Fig. 2C and D).

Total FoxO3a protein expression was not significantly affected by RE or MR (Fig. 2E). In contrast, FoxO3a Ser253 phosphorylation showed significant main effects of both RE and MR, with lower values observed across conditions and no interaction (Main effect of RE: *p* < 0.05, Main effect of MR: *p* < 0.05, Fig. 2F). Ubiquitinated proteins showed a similar pattern to total FoxO3a, with no significant main effects detected (Fig. 2G). MuRF-1 protein expression showed a significant main effect of MR, with lower values observed in the MR condition, whereas no main effect of RE was detected (Main effect of RE: *p* = 0.661, Main effect of MR: *p* < 0.05, Fig. 2H) Fbx32 protein expression was not significantly affected by either RE or MR (Fig. 2I).

## 4 Discussion

This study aimed to investigate whether the MR affects acute RE-induced muscle anabolic and catabolic signalling in rat skeletal muscles. The main results of this study were as follows: 1) 8-week MR did not suppress RE-induced muscle protein synthesis, which showed a main effect of MR, nor mTORC1 signalling, which was activated under both RE and MR conditions; and 2) the MR itself was associated with lower MuRF-1 protein expression, while FoxO3a Ser253 phosphorylation was reduced in response to both RE and MR. These results suggested that the MR for 8 weeks maintains anabolic signalling capacity and does not aggravate catabolic signalling during acute RE in rat skeletal muscle.

In this study, rats fed MR were given limited methionine through the diet. However, a higher muscle protein synthesis was observed in MR. A previous study showed that the MR for five weeks increased muscle mass, although it reduced the mixed protein and fractional rate of the cytosol at day 21 in mice [14]. Recently, the MR for broilers was shown to maintain fractional protein synthesis rate, although muscle mass decreased [31]. It appears that muscle mass does not necessarily correspond to the rate of muscle protein synthesis in skeletal muscle by MR. We assume that muscle protein synthesis is increased under malnutrition state by MR to prevent muscle mass loss caused by MR. Based on our results, the phosphorylation of AMPK, which is an intracellular energy sensor and negative regulator of mTORC1 signalling [32], was not increased by MR. Therefore, energy stress may not meditate the anabolic response in MR condition. Moreover, the finding from Longchamp et al. was reported that sulfur amino acid restriction enhanced angiogenesis in old mice via increased VEGF expression [33]. Angiogenesis is important event to enhance muscle hypertrophy in skeletal muscle [34]. Therefore, MR-induced angiogenesis may have activated muscle anabolic response in this study. However, further studies are needed to reveal how the MR regulates muscle protein metabolism.

No difference was observed in plasma methionine levels between the groups. Our results suggest that circulating methionine levels are maintained in the fasting state, even during MR. A previous study showed that the MR did not reduce plasma methionine levels, but a sulfur amino acid restriction diet, which is limited by methionine and cysteine, significantly reduced it [35]. These results suggest that lower methionine levels may be accompanied by cysteine restriction. The MR in this study included the same volume of cysteine as the normal diet. It may contribute have contributed to the methionine homeostasis. However, further studies would be required to investigate the effects MR of cysteine utilization and glutathione synthesis which are potential factors of methionine homeostasis.

Our results showed that the MR was associated with increased muscle protein synthesis and did not attenuate muscle protein synthesis or mTORC1 signalling activation after RE. The MR augmented p70S6K phosphorylation with RE. Especially, p70S6K phosphorylation has been reported as a marker of muscle hypertrophy because its activation is strongly correlated with the percentage change in muscle mass after resistance training in rat [36]. Other studies have shown that muscle contraction and exogenous amino acids stimulate p70S6K phosphorylation in skeletal muscles [2,4,7,9,24,[36], [37], [38]]. We did not observe the long-term effects of RE, such as muscle hypertrophic parameters, under MR; however, MR-induced p70S6K phosphorylation may contribute to the enhancement of muscle protein synthesis. A previous study showed that the MR did not inhibit synergistic ablation-induced muscle mass gain in mice [18]. Another study reported that the MR enhanced muscle hypertrophy induced by synergist ablation in aged mice, and that Akt Ser473 phosphorylation was also upregulated [19]. These data suggest that the MR does not necessarily negatively affect the activation of muscle anabolic responses to muscle contraction or mechanical overload.

The autophagy-lysosome and ubiquitin-proteasome systems primarily regulate protein degradation in skeletal muscle [39]. In particular, the autophagy-lysosome system is sensitive to intracellular nutritional status [40]. Previous studies have reported that the MR activates the autophagy-lysosome system in yeast [41]. However, we did not observe any changes in p62 and LC3B-II/I ratio, which are recognised markers of autophagy flux [42]. Therefore, autophagy flux was not altered under MR, even in combination with RE, in rat skeletal muscle in this study.

We showed that RE and MR reduced FoxO3a Ser253 levels, whereas MuRF1 protein expression was decreased by MR. FoxO3a is localised in the nucleus in its dephosphorylated form [43]. Its form transcripts of various genes, such as *murf-1* and *fbx32*. Therefore, our results indicated that FoxO3a translocation into the nucleus was induced by RE and MR. However, FoxO3a does not solely regulate MuRF1 protein expression, and other factors, such as NF-kB, could affect MuRF1 expression [44,45]. These findings suggested that MR-induced MuRF1 downregulation may involve FoxO3a-independent pathway in this study. Further studies are needed to elucidate the mechanism how the MR interacts with the ubiquitin-proteasome system in skeletal muscle.

3-methylhistidine is treated as a biomarker of muscle protein breakdown [3,46]. Compared to the protein expression of MuRF-1, plasma 3-methylhistidine levels were not modified by MR. This result suggested that the MR downregulated MuRF-1 protein expression but did not affect whole-body muscle protein breakdown. However, the unilateral model of RE used in this study could not completely evaluate the local effect on muscle protein breakdown in terms of 3-methylhistidine. In the future, it will be necessary to further investigate the impact of MR and RE on muscle protein breakdown using stable isotope methods.

There are several limitations in this study. Firstly, the rats were euthanized at only one time point in this study. RE modifies anabolic and catabolic signalling in skeletal muscle [2,24,37,47]. We considered 6 h after RE to be the optimal time point to capture mTORC1 signalling and muscle protein synthesis, according to previous studies [2,38]. However, setting several time points would have provided helpful knowledge to capture the comprehensive phenomenon of muscle protein metabolism and related pathway induced by MR and RE. Secondly, we did not provide direct evidence whether SAMTOR is involved with MR-induced mTORC1 activation. SAMTOR negatively regulates mTORC1 [10]. Further studies are necessary to address this point.

In conclusion, MR for 8 weeks did not impair muscle anabolic response and exacerbate catabolic response combined with RE in rat skeletal muscle. Our study would contribute to understand the impact of plant-based diets, which potentially include a lower amount of methionine than omnivorous diets, on muscle protein metabolism and the anabolic response to physical activities and exercises including RE [11].

## Funding

This research was supported by 10.13039/501100001691JSPS KAKENHI Grant Number JP21KK0177 to 10.13039/100008608SF and by JST 10.13039/501100025019SPRING, Japan Grant Number JPMJSP2101 to NF.

## CRediT authorship contribution statement

**Naoki Fukao:** Conceptualization, Data curation, Formal analysis, Funding acquisition, Investigation, Supervision, Writing – original draft, Writing – review & editing. **Mito Watanabe:** Investigation, Writing – review & editing. **Ryo Takagi:** Investigation, Writing – review & editing. **Koki Okumura:** Writing – review & editing. **Naomi Yoshii:** Investigation, Writing – review & editing. **Koma Kawabata:** Writing – review & editing. **Satoshi Fujita:** Funding acquisition, Supervision, Writing – original draft, Writing – review & editing.

## Declaration of competing interest

The authors declare that they have no conflicts of interest.

## Data Availability

The raw data can be available upon reasonable request from corresponding author.
